# Vorasidenib in IDH mutant WHO grade 2 gliomas: time to stop sitting on the fence?

**DOI:** 10.1093/noajnl/vdae003

**Published:** 2024-01-09

**Authors:** Angelo Dipasquale, Enrico Franceschi, Giuseppe Lombardi, Matteo Simonelli

**Affiliations:** Medical Oncology and Hematology Unit, IRCCS Humanitas Research Hospital, Rozzano (Milan), Italy; Department of Medical Oncology, IRCCS Institute of Neurological Sciences, Bologna, Italy; Department of Oncology, Oncology 1, Veneto Institute of Oncology IOV - IRCCS, Padua, Italy; Medical Oncology and Hematology Unit, IRCCS Humanitas Research Hospital, Rozzano (Milan), Italy; Department of Biomedical Sciences, Humanitas University, Pieve Emanuele (Milan), Italy

Approximately 2500 people per year are diagnosed with a World Health Organization grade 2 (WHO G2) isocytrate-dehydrogenase mutant (IDHm) glioma, usually at a young age (approximately 40 years) and at the height of their social and professional life.^1^ As per most recent guidelines, patients with newly diagnosed IDHm gliomas should undergo maximal safe surgical resection followed by a wait-and-see strategy or sequential radio-chemotherapy (RT-CT) depending on WHO grading and risk factors (particularly age and extent of resection).^1^ Patients having both IDHm astrocytomas and IDHm/1p-19q co-deleted oligodendrogliomas used to live long with their disease, with a median survival ranging between 3–10 years and 15–20 years, respectively.^1^ During this wide time frame, the burden of long-term side effects of their oncological treatments, such as neurocognitive deterioration from RT and fertility issues from CT, may significantly affect quality of life.^1,2^ Novel therapeutic strategies allowing clinicians to defer RT-CT in these slow-growing tumors are urgently needed.

Since the discovery of IDH mutation in a subset of gliomas, our molecular knowledge of these tumors has dramatically expanded in recent years.^1^ The mutant IDH enzyme induces a wide range of biological effects including impaired cellular differentiation and genome-wide hyper-methylation.^1,3^ Evidence from several preclinical studies demonstrated that blocking IDH pathway in both glioma cells and mouse models may elicit strong anticancer effects.^1,3^ Among small molecules, the first-in-class, brain-penetrant, dual inhibitor of the mutant IDH1-2 enzymes vorasidenib showed a favorable safety profile in a Phase I study including 52 patients with recurrent IDHm gliomas, with promising activity against nonenhancing tumors.^1,4^ The subsequent Phase III INDIGO trial randomized 331 subjects with residual or recurrent IDHm WHO G2 gliomas to receive vorasidenib or placebo.^5^ Patients must have received surgery alone in 1–5 years before (median time from surgery to randomization of 2.2–2.5 years), with no need for immediate RT-CT as for physicians’ judgment.^5^ Compared to placebo, vorasidenib significantly improved median progression-free survival (11.1 vs 27.7 months) and median time to next anticancer interventions (17.8 months vs not reached), with a tolerable safety profile.^5^ Moreover, a significant decrease in tumor growth rate has been observed in patients receiving vorasidenib (13.9% vs –2.5%).^6^ These results represent undoubtedly a breakthrough in the field of neuro-oncology but raise several significant issues that our community will face in the very close future. One of the key questions that urgently needs to be addressed is which patient should be the ideal candidate to receive vorasidenib in our daily clinical practice. Looking exclusively at the main inclusion criteria of the INDIGO trial, patients should be diagnosed with residual or recurrent IDHm WHO G2 glioma with measurable disease according to RANO low-grade glioma criteria.^5^ Most of them (82%–84%) presented a tumor dimension ≥2 cm at trial inclusion and would be formally classified as a high-risk group, therefore needing RT-CT.^1^ Although the risk estimation should be performed soon after surgery, patients recruited in the trial were considered to have an indolent disease and we may assume that their tumor size was comparable to one of the postoperative time. However, in a current scenario where “*low-risk*” cases follow a wait-and-see strategy while the “*high-risk*” receive RT-CT, several clinical features outline the potential existence of an “*intermediate-risk"* group who may be considered for vorasidenib treatment: preserved clinical condition and neurological status, no need for corticosteroids, well-controlled seizures, absence of enhancing disease, and possibly low preoperative tumor size (Table 1).^5–7^ Discussing the INDIGO study design, someone could even argue that the control group should have received RT-CT instead of placebo. Nevertheless, the trial would have failed to investigate its key secondary endpoint in case of RT-CT as a control arm: deferring other anticancer therapies and related toxicities.^5^ Undoubtedly, this will have a strong positive impact on the quality of life of patients.

**Table 1.** Potential characteristics of a patient with a diagnosis of WHO G2 IDHm gliomas suitable for treatment with vorasidenib

“*Intermediate-risk*” features
Good clinical condition (KPS 80%–100%)
Preserved neurological status
No need for corticosteroids
Well-controlled seizures
No enhancing disease on brain MRI
Low preoperative tumor volume

KPS, Karnofsky Performance Status; MRI, magnetic resonance imaging; STR, subtotal resection

Still, several other issues remain far from being solved and will nourish the debate in the coming years. First, it is still not formally known whether vorasidenib will improve OS and the crossover will not lead to a meaningful survival analysis.^5^ At least in theory, we cannot exclude changes in the biology of the tumor after vorasidenib, accelerating disease progression or leading to resistance to RT-CT. Second, the grading system in gliomas is still based on pathological features, suffering from a relevant interobserver variability in clinical practice.^8^ More reliable methods to refine grade such as methylation profile and molecular markers should be implemented to help in selecting who may benefit from vorasidenib.^9^ Third, vorasidenib should be tested in other clinical contexts, exploring its role as an “*all-comers*” strategy regardless of risk stratification for WHO G2 gliomas and as a concurrent and/or maintenance agent after RT-CT in WHO G2–4 gliomas. As for now, even its use in patients who progressed to RT-CT cannot be put aside. Fourth, the overall magnitude in PFS achieved with vorasidenib (approximately 16 months) cannot be compared to the one obtained with postoperative RT-CT in high-risk patients (more than 5 years).^1,5^ Future research should identify predictive biomarkers to tailor the use of the most appropriate postoperative treatments, such as delivering RT-CT immediately in tumors primary resistant to IDH blockade. Finally, an international effort should be implemented in revising the performance of both European Organization for Research and Treatment of Cancer and Radiation Therapy Oncology Group risk classifications in the modern era, to design a high-quality and unified prognostication system supporting our clinical decision-making.^10^ All these considerations will trigger a cascade effect, with further questions in terms of duration of therapy, long-term costs and chronic toxicities.

In conclusion, vorasidenib represents the first example of truly successful targeted therapy in IDHm glioma significantly delaying progression and use of anticancer interventions burdened with toxicities. Selecting the appropriate patient for vorasidenib should consider clinical and radiological features, as well as patients’ preferences and possibly molecular make-up, within a multidisciplinary tumor board discussion. It is time for neuro-oncologists to stop sitting on the fence and push forward the introduction of vorasidenib in the therapeutic algorithm of IDHm gliomas and in future studies addressing these still-devasting diseases.

## Authors’ statement

The text is the sole product of the author(s) and no third party had input or gave support to its writing.
